# Equine Assisted Interventions (EAIs): Methodological Considerations for Stress Assessment in Horses

**DOI:** 10.3390/vetsci4030044

**Published:** 2017-09-08

**Authors:** Marta De Santis, Laura Contalbrigo, Marta Borgi, Francesca Cirulli, Fabio Luzi, Veronica Redaelli, Annalisa Stefani, Marica Toson, Rosangela Odore, Cristina Vercelli, Emanuela Valle, Luca Farina

**Affiliations:** 1 Italian National Reference Centre for Animal Assisted Interventions, Istituto Zooprofilattico Sperimentale delle Venezie, 35020 Legnaro, Italy; lcontalbrigo@izsvenezie.it (L.C.); lfarina@izsvenezie.it (L.F.); 2 Center for Behavioral Sciences and Mental Health, Istituto Superiore di Sanità, 00161 Rome, Italy; marta.borgi@iss.it (M.B.); francesca.cirulli@iss.it (F.C.); 3 Department of Veterinary Medicine, University of Milan, 20133 Milan, Italy; fabio.luzi@unimi.it (F.L.); veronica.redaelli@unimi.it (V.R.); 4 Istituto Zooprofilattico Sperimentale delle Venezie, 35020 Legnaro, Italy; astefani@izsvenezie.it (A.S.); mtoson@izsvenezie.it (M.T.); 5 Department of Veterinary Science, University of Turin, 10095 Grugliasco, Italy; rosangela.odore@unito.it (R.O.); cristina.vercelli@unito.it (C.V.); emanuela.valle@unito.it (E.V.)

**Keywords:** equine assisted interventions, stress assessment, horse welfare

## Abstract

Equine assisted interventions (EAIs) are recently facing an increasing popularity, and are characterized by a wide diversity of practices. However, information on the welfare of animals involved in this kind of activity is often lacking. Horses are highly susceptible to work stressors related to physical constraints and/or to the need to control emotions while interacting with humans. Considerations of the emotional state of horses involved in EAIs have multiple valences: for the safety of humans and animals involved, for the quality and efficacy of interventions, as well as for ethical reasons. The aim of this unsystematic narrative review is to summarize the different approaches used for the evaluation of horses’ stress responses, investigate their application in the context of EAIs, and discuss some methodological considerations for researchers and practitioners involved in EAI. The sources of information are mostly based on electronic databases (i.e., Medline, Scopus and Google scholar), as well as on hand searches of the references of retrieved literature, and discussions with experts in the field. At present, a few studies have investigated horses’ stress responses during EAIs, and further studies are recommended, with the final aim to derive a reliable multidimensional method for assessing a horse’s reaction during therapeutic programs, ultimately helping professionals to better develop interventions by taking into consideration the animal’s perspective.

## 1. Introduction

Equine assisted interventions (EAIs) is an umbrella term that incorporates programs that aim to improve human health and wellbeing. These interventions are based upon the emotional/physical relationship that is established between the human being and the horse. They can have recreational, educative, and/or therapeutic objectives.

Reviewing the literature, a wide variety of terms describing such interventions can be found, depending on the type of intervention and context: therapeutic horseback riding, hippotherapy, equine assisted intervention, equestrian rehabilitation. Although the Federation of Horses in Education and Therapy International (HETI—formerly FRDI) made a distinction of terminology [1], terms are still used interchangeably, and this represents a source of confusion. As for the Italian context, in 2015, an agreement between the Italian government and the regional authorities was achieved on national guidelines on Animal Assisted Interventions (AAIs) [2]. These guidelines define some standards about AAIs, in particular, concerning the composition of the multidisciplinary team, the training of professionals and operators, the features of specialized centers, and the characteristics of animals, similarly to what has been established by international organizations, such as the International Association of Human Animal Interaction Organization (IAHAIO) [3]. Italian national guidelines state that only domestic species can take part in AAIs, and the horse is one out of five species (together with dog, cat, rabbit, and donkey) that are allowed to be involved in animal assisted therapy and education [2]. Actually, dogs and horses result to be the animals most frequently involved in AAIs in Italy [4], as well as worldwide [5]. Popularity of horses is linked to the long and varied history of the human-horse relationship. Over the centuries, horses progressively acquired a “mixed status” for humans: at the beginning, they represented a source of food, then were used for leisure activities and sport, or acquired the status of companion animals for others; nowadays, they can act as a co-therapist in therapeutic riding programs [6].

The variability in terms described above is associated to a great heterogeneity in approaches and methodologies among countries. At present, the world of EAIs is characterized by a wide diversity of practices; EAIs can be adapted to the human subject involved (e.g., children vs. elderly patients) and the type of disability (e.g., mental vs. physical disorders). Even the settings in which they are delivered can vary (e.g., inpatient or outpatient setting, clinical setting, home, school), as well as the specific type of activity that is planned [7].

EAIs are recently facing an increasing popularity and a rise in scientific evidence of their efficacy. Some studies have reported a variety of benefits of EAIs, in particular, in the social, emotional, physical, and educational domains [8]. Positive results have been found, for example, on children [9,10,11,12,13,14,15], adolescents [16,17], adults, and elderly, with intellectual disabilities or physical impairments [18,19,20,21,22]. Some reviews have investigated psychological outcomes [23], the effect of EAIs on physical function [24], and their efficacy as complementary interventions with children and adolescents at risk [25,26]. Notwithstanding encouraging results, some literature reviews have emphasized a number of limits in EAI research. For example, Anestis and colleagues examined the quality of the results and related outcomes from peer-reviewed research on EAIs for mental disorders, concluding that there appears to be scant justification for its use as a standalone or adjunctive treatment for any mental disorder. According to them, further studies should improve the quality of research. Most of the current studies lack a rigorous methodology (e.g., control group or consistent follow up data), are based on heterogeneous populations and small sample sizes, and moreover, do not use standardized measurement instruments [27,28].

Notwithstanding the increasing scientific interest on EAIs, few studies have tested the stress experienced by horses involved in these interventions. Horses involved in EAIs frequently work on a daily basis, and similarly to those employed for more common equestrian disciplines, can be submitted to work stressors related to physical constraints and/or ‘‘psychological’’ conflicts, such as controversial orders from the riders, or the requirement to suppress emotions [29]. In the context of EAIs, animals are also requested to relate with subjects with a variety of physical and social/emotional disabilities, although few studies have explored whether this requirement can represent an additional source of stress for the animal. As an example, subjects with physical disabilities may have balance problems, which may demand added physical strain from the animal, whereas subjects with autism spectrum disorder (ASD), instead, are characterized by persistent deficits in social communication and social interaction, and can show associated problem behaviors, such as hyperactivity, inattention, aggression, and irritability [30,31]. However, the response of animals when interacting with subjects with social and emotional problems—like those diagnosed with ASD—have not been sufficiently studied. As an example, to our knowledge, no studies have correlated any pathological behavior shown by the child with stress-related behaviors (or physiological responses) shown by the animal during EAIs. This aspect appears relevant, since the establishment of a healthy and successful relationship between the patient and the animal represents the key point for achieving therapeutic goals, and can have consequences both for the animal’s welfare and for the human’s safety.

The aim of this unsystematic narrative review is to summarize the different approaches used for the evaluation of horses’ stress responses, investigate their application in the context of EAIs, and discuss some methodological considerations for researchers and practitioners involved in EAI. This overview focuses on a subset of studies in the area chosen based on availability and author selection. The sources of information are mostly based on electronic databases (i.e., Medline, Scopus, and Google Scholar), as well as on hand searches of the references of retrieved literature, and discussions with experts in the field.

## 2. An Overview of Physiological and Behavioral Signs of Stress in the Ridden Horse

According to Etim et al. [32], animal response to stress depends on different factors, like the extent and intensity of the stressors, the animal’s previous experience, physiological status, and restraints applied. The stress reaction can be measured both at physiological or behavioral level, and a number of parameters are used as indicator of animal discomfort. An animal’s perception of the stress stimulus influences the intensity of hormonal and behavioral responses that are closely connected in a stressful context. Sources of stress for horses can be represented by physical stress induced by specific activities and being ridden, or by fear or anxiety for novel stimuli, social separation, transportation, pain and discomfort [33], or may be related to the rider’s or handler’s emotional states [34,35]. In this section, we summarize some classic and some more innovative parameters, commonly described in scientific literature as good indicators of short-term stress, such as cardiovascular parameters, cortisol, eye temperature, and behavior [33,36].

### 2.1. Physiological Measures

The two main physiological pathways involved in the activation of the stress response are the sympathetic–adrenal medulla axis and the hypothalamic–pituitary–adrenal axis (HPA). The first one is involved in the immediate response of the organism to the challenge, through the activation of the sympathetic nervous system (SNS), which results in increased heart rate, blood pressure, and secretion of the catecholamines adrenaline and noradrenaline, and reduced gastrointestinal activity. The HPA mediates longer-term effects through the release of corticotrophin releasing hormone (CRH, from the hypothalamus), that triggers the release of adrenocorticotropic hormone (ACTH) from the pituitary gland, which in turn stimulates the release of corticosteroids from the adrenal cortex [37]. Cortisol has a wide range of effects throughout the body system, since most cells express cortisol receptors. In this way, it could act on different organs, including metabolic, cardiovascular, and immune responses [38,39].

Among physiological parameters used to evaluate body adaptation responses to stressful conditions, the two abovementioned parameters (catecholamine and peripheral cortisol concentrations) represent the front-line endocrine activation to protect the body against stressful events [40], and have been widely examined in both human and veterinary medicine [41,42,43]. Catecholamines are also recognized as “stress hormones” because they are responsible for many metabolic variations, equally at rest or during exercise [44]. During the adaptive stress response, an activation of the SNS takes place, promoting fast responding mechanisms to manage a broad range of functions (e.g., respiratory, endocrine, and cardiovascular response). Catecholamines have a fundamental role also in the activation of metabolic pathways, elevating blood concentrations of glucose and free fatty acids, for the delivery of substrate to tissues. As mentioned before, the production of these hormones is a function of the increased SNS activity [45].

Adrenaline and noradrenaline in horses have been investigated, especially in various diseases and following surgery [46], exercise [45], and as a possible emotional component in equine exercise physiology [47]. Cortisol is an indicator of the extent of acute stress, and it is widely utilized in veterinary research to evaluate the short-term stress due to handling or husbandry procedures: cortisol is a time-dependent measure with a circadian rhythm that takes 10 to 20 min to reach peak values [48]. As an alternative to blood sampling, with the aim to provide a non-invasive and stress-free technique of sample collection, salivary parameter assays are being increasingly used in both human and veterinary research. Saliva offers an advantage in relation to serum or plasma, since it contains, largely, the bioavailable fraction, i.e., the fraction of the total hormone that is able to exert physiological effects [49]. Salivary cortisol is a widely used measure to evaluate stress response in horses [50,51]. In healthy horses, the time of day and season affect total cortisol concentrations, as well as body condition score, while sex and age seem to have no effects on total cortisol [52,53,54]. Among salivary parameters in human beings, the use of salivary alpha-amylase, as a marker of stress, has gained popularity during the last decade. Many studies have shown that alpha-amylase levels rise in response to both physical and psychological stress [55]. Secretion of alpha-amylase from the salivary glands is controlled by autonomic nervous signals, and several studies have revealed that salivary alpha-amylase is correlated with SNS activity under conditions of stress [56,57] in humans [58,59] and pigs [60,61]; this enzyme is also measurable in horses [55].

The major limitation of the use of blood parameters as stress indicators are the invasiveness of the approach, because of the pain and discomfort caused by venipuncture. Moreover, elevations in cortisol could be detected due to the patient’s fear of venipuncture [62], so quick procedures would be completed before cortisol levels could rise [63]. Neuro-endocrine markers may instead benefit from the ease of non-invasive saliva collection: the major limitations of salivary tests are the lack of sensitivity, and the low correlation with plasma levels [64]. Finally, both blood and saliva results could be influenced by daily fluctuations of endocrine markers [65].

Heart rate (HR) is often taken as a measure of stress in animals, since it reflects the synchronization between the vagal (which reduces HR) and the SNS (which increases HR) [66,67]. At rest, vagal regulation dominates, but with physical activity, the influence of the SNS increases. Usually HR values in the resting horse are 28–40 beats/minute (bpm) but can vary with age, breed, body weight, and associated problems [68]. Together with HR, the analysis of heart rate variability (HRV) can be applied in the evaluation of stress, including the assessment of mental stress response in horses [69]. HRV is a variation of RR, or the normal to normal (NN) interval of two adjacent QRS complexes. This variation can be analyzed with time domain analysis or frequency domain analysis [70]. Time domain analysis is used to quantify changes in RR intervals (in milliseconds) over time through the assessment of the average RR interval, the standard deviation of all RR intervals (SDNNs), and the square root of the sum of the square of differences between successive RRs (RMSSD). The RMSSD is the time domain parameter that estimates the high frequency of beat-to-beat variations that stand for the vagal regulatory activity [70]. The other method of analysis is the frequency domain analysis. It allows one to evaluate the effect of the orthosympathetic and parasympathetic system on the heart. Frequency domain analysis is based on the fact that HRV is made up of numerous oscillations that are a consequence of the action of different biological regulations that control the heart rate. In the HRV analysis, it is of special interest to evaluate the power and the frequency of the signal within certain pre-defined frequency bands. A spectral band may be subdivided into three main components: very low (VLF), low (LF), and high (HF) frequency peaks [71]. VLF bands represent the variations in the frequency which are influenced by regulatory mechanisms, such as the renin–angiotensin system and thermoregulation; LF represents the variation that is associated to the orthosympathetic/parasympathetic modulation; and HF represents variations in frequency secondary to the respiration, and is mediated by the parasympathetic nervous system [71]. HRV has been measured in different species, including horses. It has been used to monitor the response to a mental stress in association with cortisol [50,72] or with HR and selected behavioral parameters [69]. It is also useful to evaluate the response to challenging objects [73], or to assess pain associated with laminitis [74]. HRV was also recorded in horses that were actively engaged in EAI by Gehrke and colleagues [75]. To conclude, HRV analysis in horses appears to be a sensitive measure of both physical and emotional stress responses. Limitations of this measure are mainly linked to technical issues: in particular, von Borell and colleagues report that it is preferable to use a system that stores electrocardiogram (ECG) due to the characteristics of equine t-waves, and pay particular attention to the occurrence of ectopic beats and edit data accordingly [70]. Still, controversy exists regarding the question of whether or not HRV measurements provide valid information under exercising conditions, in particular, when using non-ECG-based equipment [33].

Thermography is a technique that measures the surface temperature of a body without any contact, and it is therefore very useful in detecting changes in animal’s skin temperature due to stress-induced cardiovascular changes. This technique offers the possibility to operate at distance, without interfering with the behavior and without creating any additional stress to the subject [76]. For example, Nakayama have shown a decrease in temperature in the nasal area of the macaque monkeys in the presence of a negative emotional state [77]. Other authors have worked on stress and fear responses, confirming the presence of peripheral vasoconstriction phenomena, hence a decrease in skin temperature, for example, in the ear and periocular area of rabbits, or in the tail of rats [78,79]. Studies on horses agree on the identification of the periocular area as the most useful for the relief of cutaneous temperature variations related to the presence of stress conditions [80]. The application of the thermographic technique to assess the state of the animal during potentially stressful situations should take into account the availability of reference values in basal conditions (when the animal is not stressed). Such data is often unavailable, and it is therefore necessary to test the subjects both under baseline and under stress conditions. Furthermore, infrared thermography is highly disturbed by any material between the skin and the camera, such as fur, dirt, water, and by environmental conditions, and so, requires a particularly well-thought-out experimental setup. This technique allows a massive remote inspection, even on moving subjects, but recorded skin temperatures can reflect different kinds of internal phenomena, so it is not easy to distinguish between physiological and emotional problems, or between acute and chronic diseases [76].

### 2.2. Behavioral Measures

To understand why horses, particularly stabled horses, may experience stress in their living environment, it is important to consider their ethology [81]. Wild horses are social animals living in herds, and spending much of their daily time grazing and travelling for great distances to reach the essential resources [82]. A complex social structure exists within the herd, maintained by subtle social behaviors. Horse’s domestication, which occurred during the late Neolithic period (about 5.5 thousand years ago [83]), caused these animals to live in individual or small groups within enclosures or small buildings—restricted in terms of space and movement—and to receive food and water at periodic intervals that may not be consistent with how they would have fed in the wild state [82,84]. Although some horses can well adapt to modern management through a combination of learning and experience, these environmental challenges may result in high levels of stress. In addition, many horses are requested to work on a daily basis and may thus be submitted to work stressors related to physical and emotional constraints, such as the requirement to suppress emotions [29]. By providing animals with the flexibility to respond to challenging situations, behavior represents an important mechanism of adaptation to the man-made environment, and may play an important role as an indicator of poor welfare [33]. Different categories of behavior have been pointed out as mechanisms to cope with challenging situations [85]. As an example, the influence of management factors (e.g., time spent in a box) on the risk of abnormal behavior (weaving, box-walking, wind-sucking/crib-biting stereotypies, wood-chewing) in stabled horses has been extensively studied [86].

Although it is not always easy to attribute a given behavior to a specific emotion, active avoidance behaviors (e.g., shying, bolting), as well as passive avoidance behaviors (e.g., refusal to move—when not attributable to stubbornness or laziness) has been consistently indicated as good indicators of fear/anxiety response in horse. This can be associated with behaviors such as vocalizations (e.g., blowing and snorting), ear, head, neck, and tail position and movement, retraction of the eyelids, pawing, and defecation/urination [87,88,89,90,91,92,93]. Even in the absence of fear and anxiety, the horse may experience negative mental states, such as tension and nervousness. A primary sign of negative affective states in a horse refers to his/her ear position and movement, particularly the extent to which the ears are fixed in a backward position (pinned back). Position and movement of the mouth and tongue (i.e., lip licking, lip movement, mouth opening and chomping/gaping at the bit) are also considered good indicators of discomfort in horses [92,93]. Other behaviors indicative of stress include general signs of muscle tension, unusually high or low head carriage, head and neck movements (side to side, up and down, shaking, tossing), tail movement, moving backward, or, more generally, they move in a direction not asked for by the rider [92]. It is important to notice that the absence of abnormal behavior and/or of signs of discomfort is not conclusive evidence that the horse is no longer experiencing negative mental states, but could be that he/she has been trained to remove these responses from his/her behavioral repertoire [33]. A number of studies have shown the negative consequences of inconsistent and inappropriate training methods [94,95], a process which can result in a loss of control for the horse [96,97], and which might lead, over time, to the emergence of features such as the learned helplessness response [98], and/or to depression-like conditions [99,100].

The development of a comprehensive ethogram to record the behavior of ridden horses in a range of different contexts—and of guidelines on how these behaviors should be interpreted—would provide a valuable resource for future research [93]. More studies are thus needed to evaluate and validate the ethograms currently used, and to compare information obtained with other measures, such as qualitative and or subjective behavioral assessments and physiological measures, and their association with training methods. The analysis of behavioral signs of conflict between horse and rider, and its impact on animal welfare, appears of particular importance, not only for the field of EAI, but more generally, for equestrian science [93].

## 3. Methodological Considerations for Stress Assessment during EAIs 

Few studies have investigated stress-related responses in horses involved in EAI programs, and most of them are observational studies comparing sessions with different riders and taking into consideration behavioral variables [101]. Studies evaluating the relationship between behavioral and physiological measures in assessing stress in horses have considerably increased over the last decades [42,102,103]. A recent article by Johnson et al. (2017) [104] measured stress levels of five horses working in a therapeutic riding program with military veterans with posttraumatic stress disorder (PTSD) and traumatic brain injury, who often present physical and/or psychological conditions. Plasma ACTH, glucose, serum cortisol levels, and behavior scores were determined in horses before and after therapeutic sessions with veterans, and compared to the levels of the same parameters during sessions with experienced riders. Horses ridden by veterans with PTSD did not show either physiological or behavioral stress responses, and the levels of these stress indicators remained within normal reference ranges. Kaiser et al. (2006) [101] assessed the frequency of horse’s stress related behaviors during sessions with different riders (recreational riders, individuals with physical or psychological impairment, at risk children, children with special educational needs); they concluded that being ridden by individuals with physical or psychological impairment is no more stressful than being ridden by recreational riders. Fazio et al. (2013) [105], on the other side, investigated the responses of hypothalamic–pituitary–adrenal axis during EAI with children with a physical disability and during recreational riding. In this study, endorphin and ACTH did not show significant changes after therapeutic and recreational riding sessions (in comparison with basal values), while cortisol was lower after EAI. The authors suggested that the HPA axis may be less responsive to disabled than healthy, recreational riders, although further investigation is necessary. In another study, the levels of salivary cortisol, as well as behavioral responses, during EAI and traditional hunt seat lessons or rest, were compared, and no significant changes were found [106].

The studies reviewed here often include a small sample size, and are difficult to replicate. Thus, further studies are needed to identify the most appropriate and reliable stress indicators in this context. As previously mentioned, EAIs include a wide diversity of protocols, settings and patients, therefore, standardization of research conditions represents a challenge. Moreover, information on animals employed in EAIs is often lacking, and represents a limiting factor for the subsequent interpretation of data. Therefore, a detailed description of the horse (breed, age, gender) of his/her health and physiological status, of the housing and husbandry conditions (feeding schedule, diet quality, environmental enrichment, type and amount of turnout, possibility to perform natural behavior) are mandatory. In the case of EAIs, it is of particular importance to collect information on the type of work (only therapeutic riding or also riding lessons), the daily workload for the animal, and its previous experience and training. Many horses involved in EAIs are often at the end of their career. In practice, there is the common belief that an old horse is suitable for EAIs, since is well-trained, accustomed to restrain, and less reactive to the environment; however, it is often underestimated that elderly horses can have more orthopedic problems that limit movements or make them uneven [107]. They can also be less reactive to different stimuli, since affected by other typical age diseases, like pituitary pars intermedia dysfunction (PPID) [104]. As discussed before, EAIs are performed in a variety of settings and involve different patients/clients. In many cases, such as in physical rehabilitation programs for patients with physical/motor problems, horses should be trained or accustomed to keep a certain gait, execute exercises, and to the use of specific equipment, such as horse mounting platforms, saddles and reins. There is, thus, a need for guidelines and recommendations, as well as research, on training and selection procedures for horses involved in EAIs, in order to guarantee both the animal welfare and the safety of the patients involved, and to promote the establishment of a successful human–animal interaction.

It is important to consider that during EAIs, horses are also requested to relate to different persons. In fact, EAIs are usually delivered by a multidisciplinary team that is composed by the animal handler and the medical/psychological or educational professional working in partnership [5]. Therefore, when conducting an EAI, it is necessary to also consider that transitions between successive handlers or professionals composing the team might cause distress due to the disruption of pre-existing social bonds [108]. General information on the rider also has to be considered, such as sex, age, morphometric data, experience with EAIs, and familiarity with the horse [36]. Although the development of an optimal partnership between rider and horse is the most important factor for success in many equestrian disciplines and in determining the risk of injury whilst riding, very little is known about the influence of the rider’s emotional state on that of the horses. As an example, the analysis of HRs recorded simultaneously from people and horses under different experimental handling conditions suggests the effect of human’s emotional state (i.e., nervousness) on the animal’s physiological response [35]. Further, horses are known to react differently when stroked by someone with a negative attitude to them, compared to someone with a more positive attitude [109,110]. It has also been claimed that both the rider’s and horse’s personality may affect the level of cooperation between the two [111]. This is an area of research that requires more exploration, potentially through assessing horse emotional states by means of validated ethograms and physiological measures, as well as through the analysis of the effect of personality/temperament factors within the horse–rider dyad [110]. In the case of horses involved in therapeutic activities, there is preliminary evidence suggesting the possibility that horses are sensitive to some behaviors shown by the rider, especially in the case of subjects with emotional/behavioral problems, as compared with recreational riders [101]. For example, as far as we know, no studies have examined horses’ reaction to therapeutic sessions with children diagnosed with autism, whose core symptoms affect their relational and communicative behavior, and which may show impulsivity, hyperactivity, and aggressiveness. Further studies are needed to correlate any stress-related behavior shown by the animal with the behaviors and relational style displayed by the child, in order to appraise which activities and interactions cause more discomfort in horses.

Furthermore, different kinds of work may create environmental—including social—challenges for the animal, possibly eliciting fear or anxiety responses. These challenges include, for example, being ridden alone, being asked to move independently of other horses, accepting the restraint and handling procedures imposed by the rider, being exposed to novel stimuli, situations, and environments [33,92]. Therefore, it is important to identify behavioral and/or physiological signs that indicate that the horse is either comfortable, or uncomfortable, with the activity in which it is involved [92]. In the context of EAIs, different activities, such as grooming, riding exercises, and stationary exercises, are performed by people with emotional and/or physical disorders, often under the guidance of different professional therapists and animal handlers. Creating constancy in the environment and in the signals given to the horse in various contexts can help in obtaining a consistency in his/her response. Borgi et al. [14] proposed and tested a standardized equine-assisted therapy program for children with ASD that consists in structured activities, including both work on the ground and riding. Standardization during EAI sessions can also help the animal to predict the environment and the instructions given to the horse by the animal handler or the rider. Moreover, in research, standardization of activities helps to avoid the presence of confounding variables in the comparison between different riders, both to evaluate the stress experienced by horses and the efficacy of the intervention. However, some degree of flexibility of the intervention has to be guaranteed. In fact, the course of the session is influenced, above all, by the patient’s and horse’s individuality, and by the unique interplay of the animal’s and the handler’s abilities and aptitude in relation to participant-specific goals. The role of the horse handler is crucial in detecting signs of discomfort in the horse, and consequently, in modifying or stopping the intervention if necessary.

Regarding the methodologies for horses’ stress assessment (described in Section 2), it is important to consider that in the context of EAIs, non-invasive and continuous measures should be employed to assess possible signs of stress in the animal, in order to not interfere with the therapeutic session and the spontaneous interaction between the human participant and the horse. Saliva collection for the assessment of cortisol as an indicator of stress is preferable to blood collection, which is more invasive and might itself stress the animal. Moreover, it is necessary to assess baseline levels and to consider circadian and seasonal fluctuations of hormones. Technical precautions have to be taken, also, regarding the monitoring of heart rate. The most frequently used device for horses is Polar Equine RS800cx. To use this device, it is necessary to fix it under the saddle or the lunging girth on the left side of the animals, with the positive electrode close to the withers, and the negative one in the heart area. It is essential to moisten the fur and skin with water and conductive gel; otherwise, the heart rate signal can be lost. When conductive gel is missing, even the hair’s gel can be used to maintain the adhesion of electrodes to the skin and ensure signal transmission. Any disturbance can interrupt the heart rate reception, which will result in the fall of the curve of the heart rate graph. Another useful instrument is thermography, even if many technical recommendations need to be followed to collect reliable data. The temperature variations detected are generally no more than one degree centigrade. Therefore, the measurement set up and the procedures used are essential in this kind of survey, in order to ensure the precision required [112]. Neither must be underestimated; the presence of a certain degree of variability, both due to the individual characteristics of living subjects and the sudden environmental stimuli, are present when operating on the field, in places that often are not fully controllable, like in the case of EAI settings. Thermographic reliefs should always be carried out at the same distance and angle from the subject. Very thick fur, water, dirt, or sweat on the area of interest, should be avoided. Environmental factors, such as direct solar radiation, wind, or rain, cause strong variations in cutaneous temperature; therefore, it is necessary to operate in sheltered or otherwise weather-protected structures. A good practice is to record environmental temperature and humidity present during measurements. The use of a calibrated instrument is critical, and the technical specifications must be reported; a sensor not less than 320 × 240 pixels is recommended [36,113]. It is essential to consider that an increase in cardiac work in training horses causes a temperature increase even in periocular areas, and not only in muscles. The presence of pain, or on the contrary, of positive emotional status and excitement, can generate variations in cutaneous temperature in the periocular area [76].

## 4. Conclusions and Future Perspectives

The assessment of stress-related behaviors in horses participating in EAIs, together with more objective measures to substantiate interpretations of equine behavior (e.g., physiological assessment), has both ethical and practical applications. Identifying signs of discomfort in horses represents a first step toward a more objective means of evaluating the subjective experience of horses involved in EAIs and minimizing stress during these interventions. This may contribute to promoting healthy and safe relationships between humans and animals, and avoid responses that can pose a danger to both [6,114]. Combining data from a subjective questionnaire assessment (e.g., by experienced caregivers), and objective behavioral observations and testing, can also help in assessing individual differences in adaptation strategies, and selecting horses more suitable for EAIs, as well as searching for the optimal match between the human subject involved in EAIs and the horse.

In sum, in order to assess and improve the welfare of horses involved in EAIs, evidence of the relationship between behavioral, physiological responses, and environmental factors are needed [33,36]. Future studies should use a multidimensional approach for monitoring horse welfare during both therapeutic and recreational sessions, taking into account animals’ living conditions, training style and equipment used, riding style, and the type of work performed [6]. In addition, future studies should carefully consider the possibility that the absence of conflict behavior in horses, and their compliance with trainer/handler/rider’s requests, is not always indicative of good welfare, but may be due to an apathetic state (depression-like state related to learned helplessness [98,99,100]). The final aim is to derive a reliable method for assessing a horse’s reaction during therapeutic programs, ultimately helping professionals to better develop interventions, taking into consideration the animal’s perspective.
